# Simultaneous Determination of Flavonoids, Isochlorogenic Acids and Triterpenoids in *Ilex hainanensis* Using High Performance Liquid Chromatography Coupled with Diode Array and Evaporative Light Scattering Detection

**DOI:** 10.3390/molecules18032934

**Published:** 2013-03-04

**Authors:** Bo Peng, Chun-Feng Qiao, Jing Zhao, Wei-Hua Huang, De-Jun Hu, Hua-Gang Liu, Shao-Ping Li

**Affiliations:** 1 State Key Laboratory of Quality Research in Chinese Medicine, Institute of Chinese Medical Sciences, University of Macau, Macao SAR, China; 2 School of Pharmaceutical Sciences, Guangxi Medical University, Nanning 530021, China

**Keywords:** *Ilex hainanensis*, flavonoid, isochlorogenic acid, triterpenoid, HPLC-DAD-ELSD

## Abstract

A high performance liquid chromatography coupled with diode array and evaporative light scattering detection (HPLC-DAD-ELSD) method for simultaneous determination of eight major bioactive compounds including two flavonoids (rutin and eriodictyol-7-*O*-β-d-glucopyranoside), two isochlorogenic acids (isochlorogenic acid A and isochlorogenic acid C) and four triterpenoids (ilexhainanoside D, ilexsaponin A_1_, ilexgenin A and ursolic acid) in *Ilex hainanensis* has been developed for the first time. The 283 nm wavelength was chosen for determination of two flavonoids and two isochlorogenic acids. ELSD was applied to determine four triterpenoids. The analysis was performed on an Agilent Zorbax SB-C_18_ column (250 × 4.6 mm i.d., 5 µm) with gradient elution of 0.2% formic acid in water and acetonitrile. The method was validated for linearity, limit of detection, limit of quantification, precision, repeatability and accuracy. The proposed method has been successfully applied for simultaneous quantification of the analytes in four samples of *Ilex hainanensis*, which is helpful for quality control of this plant.

## 1. Introduction

*Ilex hainanensis* Merr. (family Aquifoliaceae) is a native plant of, and mainly distributed in southern China. The leaves of *Ilex hainanensis,* named “*Shan-lü-cha”* in Chinese, are a raw material usually used as a traditional Chinese medicine (TCM) for the treatment of hyperlipidemia and hypertensive diseases [1]. Many studies have demonstrated that this herb has potential cardiovascular protective [2,3], anti-hyperlipidemic [4], anti-hypertensive [5,6], anti-inflammatory [7] and hypo-glycemic [8] effects. Phytochemical studies revealed that main components of *Ilex hainanensis* are flavonoids, triterpene glycosides and triterpene acids [9]. To date, flavonoids from *Ilex hainanensis* have been demonstrated as bioactive constituents for the treatment of hyperlipidemic diseases [10] and benign prostatic hyperplasia (BPH) [11,12]. Triterpenoids such as ilexgenin A also have a significant antibacterial activity [13]. In addition, isochlorogenic acids were also found as major components with antioxidant and antibacterial activities [14,15].

To ensure the efficacy and safety of TCMs, qualitative and quantitative analysis of their major bioactive components is very important. To date, quality control of *Ilex hainanensis* are only focused on a few individual components such as rutin [7,16,17], triterpenoids [13,18] and chlorogenic acid [19]. Therefore, it is necessary to develop a method for simultaneous quantification of multiple components in *Ilex hainanensis* because the therapeutic effects of herbs are usually derived from the integrated activity of multiple components. However, herbs may contain hundreds or even thousands of components with different structural characteristics, and a single detector may be not enough for simultaneous determination of multiple components in herbs [20]. Herein, a high-performance liquid chromatography coupled with diode array and evaporative light scattering detection (HPLC-DAD-ELSD) method was developed for simultaneous determination of eight major bioactive compounds in *Ilex hainanensis*, including two flavonoids (rutin, eriodictyol-7-*O*-β-d-glucopyranoside), two isochlorogenic acids (isochlorogenic acid A and C) and four triterpenoids (ilexhainanoside D, ilexsaponin A_1_, ilexgenin A and ursolic acid).

## 2. Results and Discussion

### 2.1. Optimization of Sample Extraction and Chromatographic Conditions

The optimization of ultrasonic extraction was performed using sample IH1. The parameters, including solvent (50%, 70% 100% methanol and ethanol), solvent volume (15 mL, 25 mL and 30 mL) and extraction time (10 min, 30 min and 60 min) were optimized by using a univariate approach. The amount of the eight investigated components was used as marker for evaluation of extraction efficiency. The optimum extraction method was as follows: solvent, methanol; solvent volume, 25 mL; ultrasonic extraction time, 30 min.

**Figure 1:**
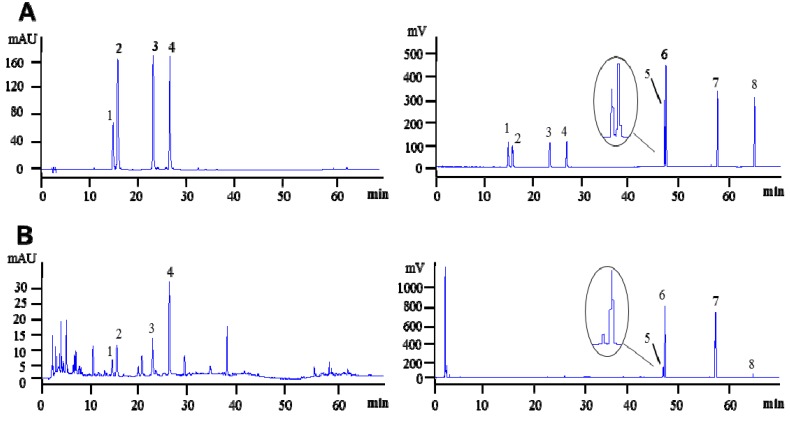
Typical HPLC-DAD-ELSD chromatograms of (**A**) mixed standards and (**B**) representative sample (IH4) detected by DAD at 283 nm (**left**) and ELSD (**right**). **1**, rutin; **2**, eriodictyol-7-*O*-β-d-glucopyranoside; **3**, isochlorogenic acid A; **4**, isochlorogenic acid C; **5**, ilexhainanoside D; **6**, ilexsaponin A_1_; **7**, ilexgenin A; **8**, ursolic acid.

After comparison with Inertsil ODS-SP (150 × 4.6 mm i.d., 5 μm) and Zorbax SB-C_8_ (150 × 4.6 mm i.d., 5 μm) columns, Agilent Zorbax SB-C_18_ (250 × 4.6 mm i.d., 5 µm) column was chosen for separation of the analytes. UV maximal absorption of flavonoids and isochlorogenic acids was at 283 nm, which was selected as the detection wavelength. Acetonitrile showed good effect on the separation of all analytes. An acidified mobile phase (0.2% aqueous formic acid) was necessary for providing satisfactory separation and peak shape for the investigated compounds in *Ilex hainanensis*. Therefore, Agilent Zorbax SB-C_18_ (250 × 4.6 mm i.d., 5 µm) column with gradient elution of 0.2% aqueous formic acid and acetonitrile was used for the separation. Typical HPLC-DAD-ELSD chromatograms are shown in Figure 1.

### 2.2. Validation of HPLC Method

The regression equations, linear ranges, LODs and LOQs of the eight analytes were determined using the developed method. As shown in Table 1, the data indicated a good relationship between the analytes’ concentrations and their peak areas within the test ranges (R^2^ ≥ 0.9991), LOD (S/N = 3) and LOQ (S/N = 10) were less than 6.88 µg/mL and 13.75 µg/mL. As shown by the results in Table 2, the overall intra- and inter-day variations (RSD) of the eight analytes were less than 1.5% and 1.8%, respectively. The repeatability (RSD, *n* = 3) at high, medium, and low levels were less than 3.2%, 3.4%, 2.4%, respectively. The analyte recoveries were between 94.6% and 103.3%, while RSD were all less than 4.1% for each analyte. Therefore, the developed HPLC-DAD-ELSD method could be considered as accurate and sensitive for quantitative determination of the eight investigated compounds.

**Table 1:** Linearity and sensitivity of analytes.

Analytes	Regressive equation	*R*^2^	Linear range (µg/mL)	LOD (µg/mL)	LOQ (µg/mL)
1	y = 4.839x − 1.603	0.9994	3.36–215.00	0.42	1.68
2	y =14.732x − 7.012	0.9993	0.83–212.00	0.21	0.83
3	y = 12.155x − 15.712	0.9994	1.69–216.00	0.42	0.84
4	y = 11.587x − 27.040	0.9993	3.13–200.00	0.39	0.78
5	y= 1.603x − 0.447	0.9993	13.75–330.00	6.88	13.75
6	y = 1.627x − 0.281	0.9991	12.56–400.00	6.28	12.56
7	y = 1.621x − 0.369	0.9991	13.13–420.00	6.56	13.13
8	y = 1.762x − 0.782	0.9992	13.06–313.50	6.53	13.06

**Table 2:** Precision, repeatability and recovery of analytes.

Analytes	Precision (RSD, %, *n* = 6)		Repeatability (RSD, %, *n* = 3)			Recovery (%, *n* = 3)	
	Intra-day	Inter-day	LL	ML	HL	Mean	RSD
**1**	0.6	1.3	2.0	0.7	2.6	98.7	2.8
**2**	0.8	1.2	2.4	3.4	1.9	97.6	1.3
**3**	0.8	1.6	2.4	1.9	1.7	96.2	2.7
**4**	1.4	1.8	2.3	3.1	1.6	100.8	0.7
**5**	0.8	1.0	1.1	1.2	2.4	94.6	3.5
**6**	0.7	0.5	1.8	1.4	2.1	103.3	0.4
**7**	0.9	1.1	2.1	2.2	2.2	98.8	4.1
**8**	1.5	1.6	2.4	2.6	3.2	98.0	2.8

*Note*: LL (low level), ML (middle level) and HL (high level) means 0.40 g, 0.50 g and 0.60 g of sample.

### 2.3. Quantitative Analysis of Investigated Compounds in Ilex Hainanensis

The developed HPLC-DAD-ELSD method was applied to simultaneous quantification of the investigated compounds in four samples of *Ilex hainanensis*. Typical chromatograms of *Ilex hainanensis* samples were shown in (Figure 1). The identification of the investigated compounds was carried out by comparison of their retention times and UV spectra (if available) with standards under the same conditions. The contents of investigated analytes in *Ilex hainanensis* are listed in Table 3. The results showed that the triterpenoids (ilexhainanoside D, ilexsaponin A_1_, ilexgenin A and ursolic acid) were the major components in *Ilex hainanensis*, which was in accordance with the previous report [13]. The contents of individual components could vary greatly due to the differences between samples. The component ratio in the extract (IH4) was very similar to that in leaves, suggesting the extract preparation method is reasonable, but the triterpenoids were significantly enriched.

**Table 3:** Contents (mg/g) of eight compounds in four samples of *Ilex hainanensis*.

Samples	Compounds (*n* = 3)								
	1	2	3	4	5	6	7	8	Tritepenoids (Sum of 5–8)
IH1	2.30	1.34	9.95	2.45	9.28	30.69	7.47	5.64	53.08
IH2	1.14	0.26	3.41	1.22	8.75	22.05	23.32	5.08	59.20
IH3	1.13	0.51	3.18	1.46	7.72	18.23	16.07	3.95	45.97
IH4	2.52	2.12	2.56	5.91	17.62	41.17	50.57	10.99	120.35

IH1–3 were leaves, IH4 was 70% ethanol extract of the leaves.

## 3. Experimental

### 3.1. Chemical Materials and Reagents

**Figure 2:**
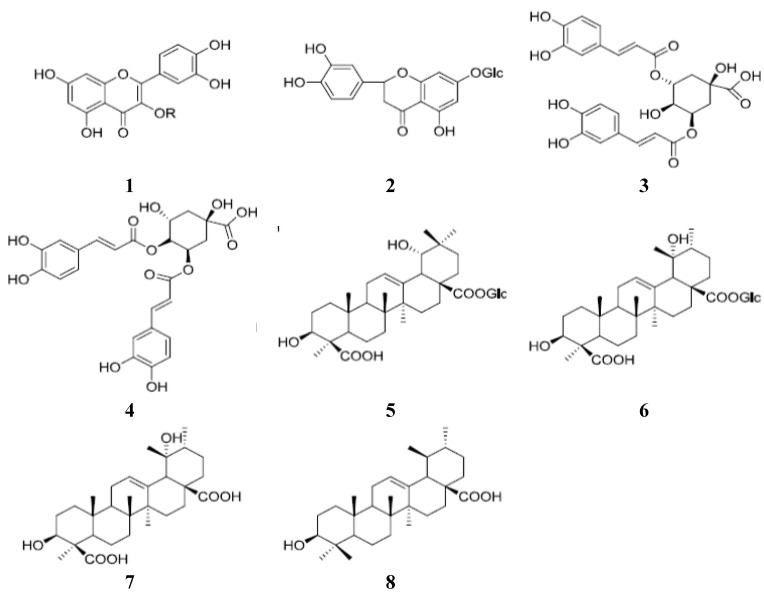
Chemical structures of the eight investigated compounds.

Four samples of *Ilex hainanensis*, leaves (IH1–3) and its extract (IH4), were provided by Guilin Otigh Natural Medicine Company Limited (Guangxi Zhuang Autonomous Region, China). The botanical origin of samples was confirmed by Prof. Huagang Liu, one of the corresponding authors. The voucher specimens were deposited at the Institute of Chinese Medical Sciences, University of Macau, Macao, China. Rutin (**1**), isochlorogenic acid A (**3**), isochlorogenic acid C (**4**) were purchased from Pureone Biotechnology Company (Shanghai, China). Eriodictyol-7-*O*-β-d-glucopyranoside (**2**), ilexhainanoside D (**5**), ilexsaponin A_1_ (**6**), ilexgenin A (**7**), ursolic acid (**8**) were isolated in our laboratory (Figure 2). The structures were confirmed by comparing their MS and NMR data with literature data [21,22,23,24]. All the purities were more than 98% as determined by HPLC-DAD-ELSD. Acetonitrile and formic acid were HPLC-grade from Merck (Darmstadt, Germany) and deionized water was purified by a Milli-Q purification system (Millipore, Bedford, MA, USA).

### 3.2. Sample Preparation

Dried sample powder (0.5 g) was extracted with methanol (25 mL) in an ultrasonic cleaning bath (Branson Ultrasonic Corporation, Danbury, CT, USA). Ultrasonication (44 KHz, 250 W) was performed for 30 min at room temperature. After centrifugation for 10 min at 2,000 × *g*, 2 mL of supernatant was transferred into a 5 mL volumetric flask and made up to its volume, and then filtered through 0.22 µm nylon membrane filter (Whatman, Maidstone, UK) before injection into HPLC system. Each sample was prepared in duplicate for quantification of analytes.

### 3.3. HPLC-DAD-ELSD Analysis

All analyses were performed on an Agilent Series 1200 system (Agilent Technologies, Santa Clara, CA, USA), consisting of a vacuum degasser, a quaternary pump, an autosampler, and a diode-array detector, controlled by Agilent 1200 LC Software. Separation was performed on an Agilent Zorbax SB-C_18_ column (250 × 4.6 mm i.d., 5 µm) at 30 °C. The flow rate was set at 1.0 mL/min and sample injection volume was 5 µL. The analytes were separated at gradient elution system consisted of (A) 0.2% formic acid in water and (B) acetonitrile as mobile phases. The gradient was as follows: 0–15 min, 15–18% B; 15–25 min, 18–23% B; 25–35 min 23–30% B; 35–40 min 30–40% B; 40–50 min 40–50% B; 50–55 min 50–80% B; 55–60 min 80–100% B; 60–70 min 100% B, and finally, reconditioning the column with 15% B isocratic for 5 min. Two flavonoids and two isochlorogenic acids were detected at 283 nm. ELSD was applied to determine the four triterpenoids. The drift tube temperature for ELSD (Alltech ELSD 3300ES, Alltech Associates, Deerfield, IL, USA) was set at 50 °C, and the nebulizing gas flow rate was 1.5 L/min, gain value was 8.

### 3.4. Calibration Curves

Stock solution was prepared by weighing the eight reference compounds accurately and dissolving them in methanol. Then the stock solution was diluted to appropriate concentrations for construction of calibration curves. For each compound, at least six concentrations of the solution were analyzed in duplicates. For compounds **1**–**4** the calibration curves were constructed by plotting the peak area (UV signal) versus the concentration of each analyte, while for compounds **5**–**8**, calibration curves were constructed by plotting the logarithmic value of the peak area (ELSD signal) versus logarithmic value of the concentration.

### 3.5. Limits of Detection and Quantification

The stock solution with eight reference compounds was diluted to a series of appropriate concentration with methanol, and an aliquot of the diluted solution were injected into HPLC for analysis. The limits of detection (LOD) and quantification (LOQ) were determined based on the signal-to-noise ratio (S/N) of about 3 and 10, respectively.

### 3.6. Precision, Repeatability and Accuracy

Intra- and inter-day variations were applied to measure the precision of the developed method. For intra-day variability test, the solution of sample IH1 was injected for six times within one day, while for inter-day variability test, the solutions were analyzed in duplicates for consecutive three days. The relative standard deviation (RSD) was utilized as a measurement for intra- and inter-day.

The repeatability of the developed method was measured at three levels (0.40 g, 0.50 g, 0.60 g) of sample IH1. The samples of each level were extracted and analyzed triplicates as the developed method. The recovery test was performed by adding known amount of individual standards into certain amount (0.25 g) of sample IH1, The mixtures were extracted and analyzed using the developed method mentioned above. Three replicates were prepared for the test.

## 4. Conclusions

An HPLC-DAD-ELSD method was developed for simultaneous determination of eight compounds, including two flavonoids, two isochlorogenic acids and four triterpenoids, in *Ilex hainanensis*, which should be helpful to control the quality of *Ilex hainanensis*.
